# Refining the genetic architecture of flag leaf glaucousness in wheat

**DOI:** 10.1007/s00122-019-03522-x

**Published:** 2020-01-17

**Authors:** Tobias Würschum, Simon M. Langer, C. Friedrich H. Longin, Matthew R. Tucker, Willmar L. Leiser

**Affiliations:** 1 State Plant Breeding Institute, University of Hohenheim, 70593 Stuttgart, Germany; 2 School of Agriculture, Food and Wine, University of Adelaide, Waite Campus, Urrbrae, SA Australia; 3 Present Address: BASF Agricultural Solutions GmbH, Gatersleben, Germany

## Abstract

**Key message:**

The cuticle is the plant’s barrier against abiotic and biotic stresses, and the deposition of epicuticular wax crystals results in the scattering of light, an effect termed glaucousness. Here, we dissect the genetic architecture of flag leaf glaucousness in wheat toward a future targeted design of the cuticle.

**Abstract:**

The cuticle serves as a barrier that protects plants against abiotic and biotic stresses. Differences in cuticle composition can be detected by the scattering of light on epicuticular wax crystals, which causes a phenotype termed glaucousness. In this study, we dissected the genetic architecture of flag leaf glaucousness in a panel of 1106 wheat cultivars of global origin. We observed a large genotypic variation, but the geographic pattern suggests that other wax layer characteristics besides glaucousness may be important in conferring tolerance to abiotic stresses such as heat and drought. Genome-wide association mapping identified two major quantitative trait loci (QTL) on chromosomes 3A and 2B. The latter corresponds to the *W1* locus, but further characterization revealed that it is likely to contain additional QTL. The same holds true for the major QTL on 3A, which was also found to show an epistatic interaction with another locus located a few centiMorgan distal to it. Genome-wide prediction and the identification of a few additional putative QTL revealed that small-effect QTL also contribute to the trait. Collectively, our results illustrate the complexity of the genetic control of flag leaf glaucousness, with additive effects and epistasis, and lay the foundation for the cloning of the underlying genes toward a more targeted design of the cuticle by plant breeding.

**Electronic supplementary material:**

The online version of this article (10.1007/s00122-019-03522-x) contains supplementary material, which is available to authorized users.

## Introduction

**Fig. 1:**
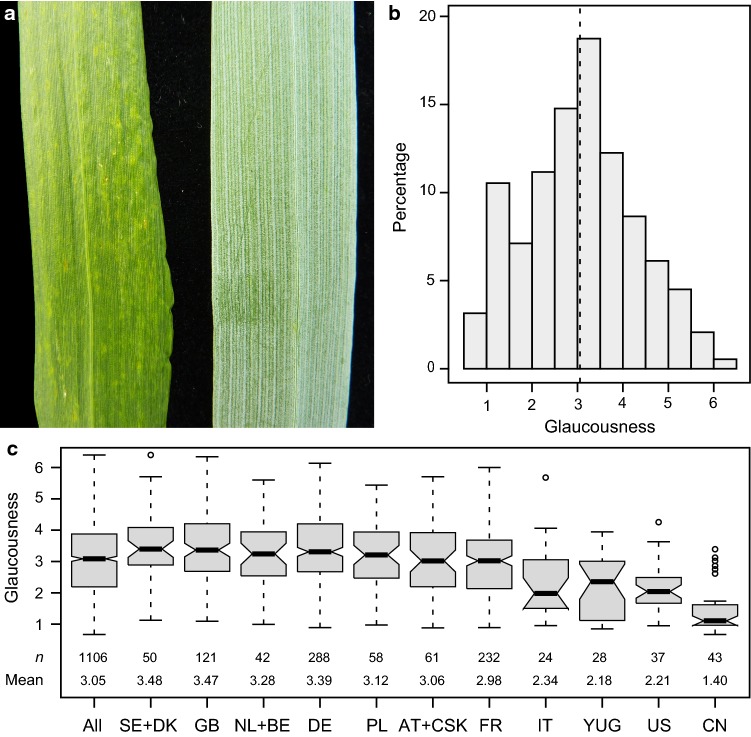
Flag leaf glaucousness in wheat. **a** Examples of a glossy and a glaucous flag leaf. **b** Histogram of flag leaf glaucousness in the panel of 1106 winter wheat cultivars. **c** Boxplots showing glaucousness dependent on the cultivars’ country of origin. *AT* Austria, *BE* Belgium, *CN* China, *CSK* former Czechoslovakia, *DE* Germany, *DK* Denmark, *FR* France, *GB* Great Britain, *IT* Italy, *NL* The Netherlands, *PL* Poland, *SE* Sweden, *US* United States of America, *YUG* former Yugoslavia, Serbia, Croatia

The plant cuticle is at the surface of aerial plant organs and thus represents the plants’ outermost point of interaction with their environment. It is an extracellular matrix consisting of a lipophilic cuticular layer and epicuticular depositions that form a continuous hydrophobic sheet on epidermal cell walls (von Wettstein-Knowles 1976; Samuels et al. 2008; Yeats and Rose 2013; Adamski et al. 2013). Glaucousness refers to the bluish-silverish-gray appearance of organs, such as the flag leaf, stem or spikes, and is caused by the scattering of light due to deposition of wax crystals on the plant’s surfaces (Fig. 1a). The opposite form is referred to as non-glaucous or glossy. In wheat, the glaucous appearance is due to the presence of β-diketones. Only glaucous organs show an accumulation of tubular/rod-shaped wax structures typical for β-diketone-rich wax, whereas glossy organs are completely devoid of any visible wax protruding from the surface (Adamski et al. 2013; Hen-Avivi et al. 2016). Glaucousness has been associated with several traits and physiological processes, mainly related to an increased drought and heat tolerance and thus higher yield under dry conditions (e.g., Johnson et al. 1983; Richards et al. 1986; Febrero et al. 1998; Samuels et al. 2008; Bi et al. 2017).

Early genetic studies in wheat revealed two loci for wax production, termed *W1* and *W2*, and two *Inhibitor of wax* loci, *Iw1* and *Iw2*, that inhibit glaucousness (Tsunewaki and Ebana 1999). *W1* and *Iw1* are located on chromosome 2B and *W2* and *Iw2* on chromosome 2D. Both wax production loci *W1* and *W2* can produce a glaucous appearance. The two wax inhibitor loci act dominantly and the presence of either one is sufficient to inhibit *W1* and/or *W2* and thus result in a glossy phenotype. Adamski et al. (2013) showed that *Iw1* inhibits the formation of β-diketones and hydroxyl-β-diketones in the cuticle. Further analyses revealed the genetic control of glaucousness to be more complex, as several quantitative trait loci (QTL) were identified (Börner et al. 2002; Kulwal et al. 2003; Mason et al. 2010; Bennett et al. 2012). The A genome progenitor *Triticum urartu* is non-glaucous and lacks β-diketones, and only the B and D genomes were thought to contain major glaucousness loci (Tsunewaki and Ebana 1999). Interestingly, Bennett et al. (2012), studying a biparental population of two Australian wheat lines, identified a major QTL on chromosome 3A that accounted for 5–49% of the genetic variation in glaucousness.

The biosynthesis of major cuticle components consists of three major steps within epidermal cells, starting with the de novo synthesis of C_16_ fatty acids in plastids. These are converted into acyl-CoAs, which are exported to the endoplasmic reticulum before being further elongated to very-long-chain C_20_–C_34_ fatty acyl-CoAs. These can become free fatty acids or be further modified by either the alcohol-forming pathway resulting in the formation of even-numbered primary alcohols and alkyl esters, by the alkane-forming pathway leading to secondary alcohols, aldehydes, ketones and odd-numbered alkanes, or by the β-diketone pathway (Hen-Avivi et al. 2016; Bi et al. 2017). The latter has been proposed in barley based on extensive genetic and biochemical studies of *eceriferum* (*cer*) mutants and proposed to include three enzymatic steps (von Wettstein-Knowles 2012). However, it had remained unclear whether β-diketone biosynthesis is controlled by three very tightly linked genes (*Cer*-*c*, -*q* and -*u*) or by a single gene encoding a multifunctional protein. Schneider et al. (2016) recently resolved this and showed that the *Cer*-*cqu* locus consists of a cluster of three genes. The *Cer*-*cqu* locus is in a syntenic position to the wheat *W1* locus (Varshney et al. 2006), and Hen-Avivi et al. (2016) showed that both the *W1* and the *Cer*-*cqu* loci incorporate similar metabolic gene clusters required for β-diketone biosynthesis. Both comprise genes encoding three types of enzymes: chalcone synthase-like type-III polyketide synthase (PKS), putative lipase/carboxylesterase or hydrolase (HYD) and cytochrome P450 (P450). In wheat, the locus appears to be highly dynamic resulting in several duplications of some of those genes. In addition, a putative wax ester synthase (WES), belonging to the membrane-bound *O*-acyl transferase family, is also located in the *W1* cluster.

Moreover, the recently identified *W3* locus (Zhang et al. 2015) is linked to *W1* and also involved in β-diketone biosynthesis, suggesting that it may be part of the identified metabolic cluster. Hen-Avivi et al. (2016) also showed that the dominant wax inhibitor *Iw1* is not only genetically close to *W1*, as both loci were completely linked to the same marker, but also physically close. However, the genomic interval between flanking markers, particularly the *W1* locus, was found to be completely different between a glaucous and a glossy emmer wheat. Huang et al. (2017) recently showed that *Iw1* encodes a long noncoding RNA (lncRNA) from which a miRNA is produced that targets and down-regulates a carboxylesterase within the *W1* locus. A similar sequence was found on chromosome 2D at the location to which *Iw2* has been fine-mapped. Thus, *Iw1* and *Iw2* produce miRNAs that repress expression of the carboxylesterase gene at the *W* loci required for the production of β-diketone waxes, indicating a key role of the carboxylesterase gene(s). Interestingly, Huang et al. (2017) also observed a down-regulation of other genes from the *W1* locus, suggesting that there is likely another mechanism inactivating multiple genes in this region.

In this study, we revisited the genetic control underlying flag leaf glaucousness in wheat by performing association mapping in a large panel of 1106 wheat cultivars of global origin. We identified two major QTL on chromosomes 3A and 2B. The 2B QTL corresponds to the *W1*/*Iw1* locus but fine-mapping revealed it to be more complex, likely comprising several QTL. Two potentially homoeologous putative QTL were found on chromosomes 2A and 2D. The 3A QTL may also be complex and in addition was found to display an epistatic interaction with another locus located ~ 6 cM apart. Collectively our results expand our understanding of the genetic architecture of flag leaf glaucousness and lay the foundation for the cloning of the underlying genes.

## Materials and methods

### Plant material and experimental design

This study is based on a panel of soft winter wheat (*Triticum aestivum* L.) cultivars that has been described previously (Boeven et al. 2016a; Würschum et al. 2015, 2017a, 2018a, b). It includes 1106 cultivars from worldwide origin released during the past decades, but with a focus on cultivars from Europe. The test locations were Hohenheim (HOH, 48°42′54.4″ N, 9°11′22.6″ E, 400 m above sea level (asl)), Ihinger Hof (IHO, 48°44′42.6″ N, 8°55′30.8″ E, 493 m asl) and Oberer Lindenhof (OLI, 48°28′25.5″ N, 9°18′17.9″ E, 700 m asl). The experiment was conducted in the 2012/2013 growing season in a partially replicated design with a replication rate of 1.25 per location (Williams et al. 2011). Entries were sown in observation plots of two rows and 1.25 m length.

Glaucousness was assessed on flag leaves on a 1–6 scale (Fig. S1a). A score of 1 was given when no glaucousness was visible on the abaxial and adaxial sides of the flag leaves, 2 for a light glaucousness on the abaxial side, 3 for a strong glaucousness on the proximal half of the abaxial leaf surface, 4 for a strong glaucousness on the entire abaxial leaf surface, 5 for a strong glaucousness on the entire abaxial and half of the adaxial leaf surface, and 6 refers to fully glaucous flag leaves, i.e., the entire abaxial and adaxial leaf surfaces show a strong glaucousness.

Phenotypic data were analyzed as described previously (Würschum et al. 2017b). In brief, best linear unbiased estimates (BLUEs) were estimated across all three locations, assuming fixed effects for the genotype. Heritability (*h*^*2*^) was estimated following the approach suggested by Piepho and Möhring (2007). All statistical analyses were performed using the statistical software R (R Development Core Team 2014) and ASReml-R 3.0 (Gilmour et al. 2009).

### Genotypic and genomic analyses

All lines were genotyped by genotyping-by-sequencing (GBS) at Diversity Arrays Technology (Yarralumla, Australia) using the Wheat GBS 1.0 assay (DArTseq). Markers with a minor allele frequency < 0.05 were removed resulting in a total of 44,500 markers, of which for 23,720 markers a genetic map position was available (Li et al. 2015). The CloneIDs of the silico DArT markers were given a ‘D’ and that of the SNP markers a ‘S’ prefix. The physical positions of the markers were taken from the wheat reference genome sequence IWGSC RefSeq v1.0 (The International Wheat Genome Sequencing Consortium 2018).

Three carboxylesterase/hydrolase (HYD) genes (TraesCS2B01G006100, TraesCS2B01G006500 and TraesCS2B01G007100) located at the *W1* locus were sequenced by Sanger sequencing. The identified polymorphisms were converted into KASP markers (Fig. S13). All KASP assays were run using the standard KASP PCR conditions (https://www.lgcgroup.com/LGCGroup/media/PDFs/Products/Genotyping/KASP-thermal-cycling-conditions-all-protocols.pdf). In addition, a TaqMan^®^ assay was developed to assess copy number variation of TraesCS2B01G006100 and TraesCS2B01G006500. The TaqMan^®^ probe was designed to ensure a very high specificity to these two genes and the forward and reverse primers to bind only to the two target genes. *TaCO2* was used as reference gene, and the assay was run with four replications per genotype (Würschum et al. 2017a). All primer sequences and PCR conditions can be found in Supplementary Table S8.

For association mapping, an additive genetic model was chosen and mapping was done with a mixed model incorporating a kinship matrix as described previously (Würschum et al. 2017b). To control for multiple testing, a Bonferroni-corrected threshold of *P* < 0.05 was applied. The total proportion of genotypic variance (*p*_G_) explained by the detected QTL was calculated by fitting the significantly associated markers in the order of the strength of their association simultaneously in a linear model. The ratio *p*_G_ = $R_{\text{adj}}^{2}$/*h*^*2*^, where $R_{\text{adj}}^{2}$ refers to the adjusted $R^{2}$ from the linear model and *h*^*2*^ to the heritability of the trait, yielded the proportion of genotypic variance (Utz et al. 2000). The *p*_G_ values of individual QTL were accordingly derived from the sums of squares of the QTL (SS_QTL_) in this linear model. The allele substitution (α) effects were derived as the regression coefficient from models with only the marker under consideration.

Genomic prediction was done by Ridge Regression-BLUP employing the R package ‘rrBLUP’ (Endelmann 2011) and fivefold cross-validation. In addition, identified QTL were included as fixed effects in the model (Boeven et al. 2016b). The prediction ability of either QTL-based or genomic prediction was estimated as the Pearson’s correlation coefficient between observed and predicted trait values.

## Results

### Phenotypic variation of flag leaf glaucousness in winter wheat

The analysis of flag leaf glaucousness in a panel of 1106 winter wheat cultivars revealed a large amount of variation, ranging from glossy, non-glaucous to fully glaucous flag leaves with the entire abaxial and adaxial leaf surfaces showing strong glaucousness (Fig. 1a, b, Table S1). The genotypic variance of flag leaf glaucousness and the genotype-by-location interaction variance were both significant. However, the ratio between the two was 10:1, indicating a much stronger influence of the genotype on the expression of glaucousness than its interaction with the environment. Consistent with this, the heritability was high at 0.87.

For most of the cultivars, the country of origin was known, and we therefore analyzed flag leaf glaucousness dependent on the geographic origin (Fig. 1c). While there was no real difference between most European countries, cultivars from the southern European countries Italy and former Yugoslavia, as well as from the USA and China were on average the least glaucous. This was surprising, as owing to the presumed role of glaucousness in conferring drought and heat tolerance, an increasing glaucousness toward lower latitudes with their warmer and dryer climates might have been expected. While this may be an artifact of panel composition, it does not appear to be linked to cultivars of different ages representing the different geographic origins (Fig. S1b). Alternatively, it may also indicate that glaucousness is less influential in conferring tolerance to these abiotic stresses. On the other hand, the variation observed among cultivars from within all other countries was rather large, covering the entire phenotypic range. This may simply reflect the variation present in breeding germplasm without any selection pressure, but may also indicate a natural or artificial selection due to an advantage of either glaucous or glossy types under certain environmental conditions not specific to geographic origins.

### Deciphering the genetic architecture of flag leaf glaucousness

**Fig. 2:**
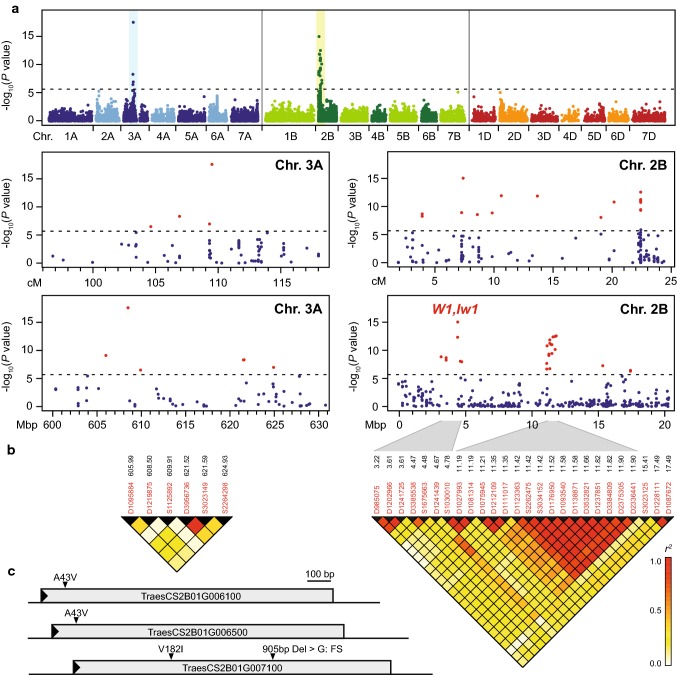
Identification of glaucousness QTL. **a** Manhattan plot showing the results from the genome-wide scan for flag leaf glaucousness. The dashed horizontal line indicates the significance threshold (Bonferroni-corrected *P* < 0.05). The regions of the two major QTL on chromosomes 3A and 2B are shown based on the genetic map and physical positions of the markers (IWGSC RefSeq v1.0). **b** Linkage disequilibrium (*r*^*2*^) among the significantly associated markers. **c** Polymorphisms in the three carboxylesterase/hydrolase (HYD) genes at the *W1* locus. The genes are shown with 5′ and 3′ UTR

To dissect the genetic control underlying the variation in flag leaf glaucousness, we performed genome-wide association mapping (Table S2). All lines were genotyped by a genotyping-by-sequencing approach yielding 44,500 polymorphic markers, with 23,720 of them having a known genetic map position (Li et al. 2015). The genome-wide scan yielded 40 significant (Bonferroni-corrected *P* < 0.05) marker-trait associations that all stem from the two major peaks on chromosomes 3A and 2B discernible in the Manhattan plot (Fig. 2a, Tables 1, S3, S4). Additional putative QTL were defined as peaks in the Manhattan plot that reached just slightly below the significance threshold. Four of these putative QTL were identified on chromosomes 2A and 2D, as well as on 6A and 7B. The proportion of genotypic variance explained jointly by all QTL amounted to 46.4%. Individually, a marker for the QTL on chromosome 3A explained 13.5% and thus the largest proportion of the genotypic variance, followed by a marker for the QTL on 2B with 6.8%. Notably, even after correction for collinearity by a joint fit of all markers in a linear model, other markers besides these two also captured genotypic variance from the 3A and 2B QTL. This indicated that the two major QTL identified here for flag leaf glaucousness may genetically be more complex. Interestingly, the additional putative QTL explained between 3.7 and 6.1% of the genotypic variance. The genetic map and physical positions of the putative QTL on 2A and 2D suggest that they may be homoeologous to the major QTL on chromosome 2B (Figs. S2, S3).

**Table 1:** Mapped markers identified as significantly associated with flag leaf glaucousness in winter wheat

Marker	Chr.	Pos. (cM)	Pos. (bp)^c^	*P* value	*p*_G_^a^	Effect	*p*^d^
S1125892	3A	104.6	609,909,609	3.1e−7	0.12	− 0.06	0.89
S3023149	3A	106.9	621,586,759	4.6e−9	0.77	0.41	0.23
D3956736	3A	106.9^b^	621,515,670	4.6e−9	0.06	0.40	0.24
S2264298	3A	109.3	624,928,879	1.1e−7	3.19	− 0.39	0.39
D1219875	3A	109.5	608,499,059	2.6e−18	13.48	0.53	0.19
D1095884	3A	109.5^b^	605,990,824	8.0e−10	0.48	− 0.38	0.23
D1116323	2B	7.3	–	1.2e−9	2.61	0.32	0.43
S1675663	2B	7.4	4,482,655	8.9e−16	6.79	− 0.28	0.51
D1212583	2B	8.6	–	2.8e−9	0.26	− 0.21	0.67
D1075945	2B	20.2	11,207,595	1.7e−11	0.01	0.23	0.62
D2336441	2B	22.4	11,898,548	2.8e−13	0.08	0.27	0.63
D1228111	2B	24.7	17,493,486	3.8e−7	1.79	− 0.07	0.49
S3023125	2B	40.7	15,410,148	5.4e−8	0.11	0.38	0.29
*Additional putative QTL*^e^							
D2258266	2A	33.7	2,731,273	5.1e−6	3.57	− 0.25	0.61
S983407	6A	92.5	283,030,630	3.3e−5	4.22	− 0.60	0.90
D1092286	7B	196.0	701,866,202	6.9e−6	6.08	− 0.34	0.64
D1113129	2D	5.8	2,873,572	8.0e−6	3.77	− 0.42	0.77

For chromosome 2B, one marker for each of the seven LD blocks (Fig. S5b) was used for this analysis

^a^Proportion of explained genotypic variance

^b^Unmapped marker placed on the genetic map based on LD with mapped markers

^c^Physical positions of the markers based on IWGSC RefSeq v1.0

^d^Frequency of the allele increasing glaucousness

^e^Not significant at the Bonferroni-corrected significance threshold of *P* < 0.05

**Fig. 3:**
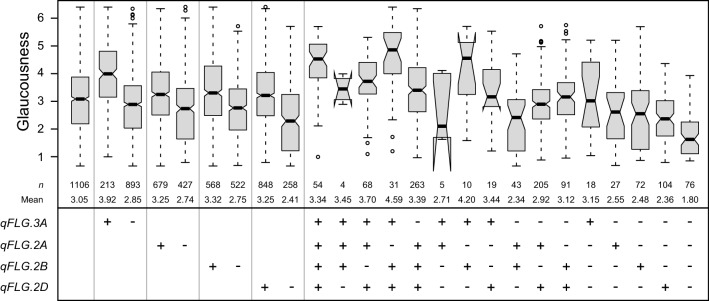
Effects of flag leaf glaucousness QTL. Boxplots showing the effects of individual QTL (*qFLG*), as well as combinations thereof on flag leaf glaucousness. *qFLG.3A*, D1219875; *qFLG.2A*, D2258266; *qFLG.2B*, S1675663; *qFLG.2D*, D1113129

The allele substitution effect of the QTL on chromosome 3A was approximately 0.5 and that of the 2B QTL approximately 0.3 (Table 1). Genotypes carrying the glaucousness-increasing allele at these two QTL were generally among the most glaucous, and glaucousness decreased with fewer glaucousness-increasing alleles at the 3A, 2B, 2A and 2D QTL (Fig. 3). This indicates that the effects of these glaucousness loci are at least to some extent additive.

The identified QTL explained approximately half of the genotypic variance, indicating that the remainder might be attributable to epistatic effects or to QTL with effects too small to be detected in association mapping. We therefore performed a genome-wide prediction, an approach that estimates effects for all markers simultaneously and thereby also captures the effects of small-effect QTL. The prediction ability of this genome-wide approach was substantially higher (mean ~ 0.75) than that based solely on identified QTL (mean 0.42 for the 3A and 2B QTL, and 0.58 for the 3A and 2B QTL plus four putative QTL), illustrating that additional small-effect additive genetic loci also contribute to the genetic architecture of flag leaf glaucousness (Fig. S4). Taken together, our analyses revealed that flag leaf glaucousness is a complex trait that besides small-effect loci is controlled by some medium- to major-effect QTL.

### The major QTL on 2B is the *W1* locus

The significantly associated markers from chromosome 2B cover a region approximately 20 cM in length without a clearly defined peak (Fig. 2a). The same markers plotted according to their physical position, however, revealed two possibly even four peaks, indicating this locus to be more than a single gene. This was further substantiated by the analysis of linkage disequilibrium (LD) among these markers, which revealed blocks of markers in high LD, but rather low LD among these blocks (Figs. 2b, S5, S6). Genome analysis revealed that the region on chromosome 2B between 3 and 5 Mbp corresponds to the *W1* locus, harboring the P450, PKS, HYD and WES genes (Fig. S7). Notably, the seven significant markers from that region were not all in high LD. This might be due to some of them being actually associated with *Iw1*, or to the complexity of the *W1* locus with several genes affecting wax synthesis. Exome capture data from 31 cultivars revealed no polymorphism in the WES gene, but in one of the carboxylesterase/hydrolase (HYD) genes located ~ 10.5 kb from the most strongly associated marker S1675663. We therefore sequenced all three HYD genes to assess these primary targets of the *Iw1* miRNA for polymorphisms. This revealed the same polymorphism (A43V) in TraesCS2B01G006100 and TraesCS2B01G006500, while one polymorphism resulting in an amino acid change (V182I) and a 1 bp InDel resulting in a frameshift were found in TraesCS2B01G007100 (Figs. 2c, S7). We genotyped a subset of 185 lines for these four polymorphisms and found them to be in high LD with each other as well as with the two most strongly associated markers from the *W1* region (Fig. S8). The two genes TraesCS2B01G006100 and TraesCS2B01G006500 are completely identical in their coding sequence and also the A43V polymorphism was in complete LD, indicating a gene duplication with the mutation resulting in the polymorphism predating the duplication event. Accordingly, the four polymorphisms each explained a comparable proportion of genotypic variance, and consequently, none of them can be ruled out as being causal (Table S5). Their explained genotypic variance was slightly higher than that of the two markers, which may indicate that they are closer to the causal polymorphism(s), but may also be due to a more accurate genotyping as compared to the genome-wide marker data.

Intrigued by the potential duplication of TraesCS2B01G006100 and TraesCS2B01G006500, we designed a TaqMan^®^ assay targeting both genes, in order to assess them for copy number variation (CNV). The assay revealed no clearly defined copy number variants, but the ratio of the target genes to the internal control gene explained 5.85% of the genotypic variance in the same subset of 185 lines (Table S5). This is unlikely for an artifact produced by the assay and consequently indicates that there is copy number variation of these two HYD genes. As for the identified polymorphisms in the three HYD genes, it is not possible to determine if the presumed copy number variation is causal for the effect of this locus or not. Collectively, these results corroborate the complex nature of the *W1* locus with several polymorphisms in different genes likely involved in wax biosynthesis, several resulting haplotypes, as well as potentially copy number variations of some of these genes. Thus, whether the identified polymorphisms are causal and contribute to natural variation in flag leaf glaucousness in wheat cannot be unambiguously determined with a diversity panel and requires further research on the molecular level.

Another group of significantly associated markers comprises 16 markers located between 11 and 12 Mbp (Fig. 2a, b). However, it was not possible to further pinpoint this QTL and genome analysis revealed no obvious candidates. Two further potential peaks in the Manhattan plot are at around 15 and 17 Mbp with one and two significant markers, respectively, and several P450 genes are located in those regions. In summary, the major QTL identified on chromosome 2B represents the *W1* locus, but resolved into at least one additional locus distal to *W1*. Further work on a genetic and molecular level is required to confirm the presence of these putative QTL and to identify the underlying genes contributing to glaucousness in wheat.

### Characterization of the major QTL on 3A

The second major QTL was identified on chromosome 3A (Figs. 2a, S9). Bennett et al. (2012) recently reported a major glaucousness QTL in a biparental population, located on chromosome 3A close to the SSR marker *wmc264*. The physical position of this SSR marker is at 625.8 Mbp and thus in the same chromosomal region as the QTL identified here, indicating that they are identical. Most of the six significantly associated markers, however, were in low LD with each other, with at least two of them capturing a substantial proportion of genotypic variance in a joint fit (Fig. 2b, Table S3). Such a pattern can be explained in several ways. It is possible that there is indeed only a single gene underlying the QTL, but in the absence of a marker in high LD with the causal polymorphism and a more complex LD structure in the region, several markers could capture the variance of that gene. Alternatively, copy number variation has also been shown to result in this kind of pattern, as single biallelic markers cannot fully capture the variance of a multiallelic QTL (Würschum et al. 2017a). Another explanation is that, as observed for the 2B QTL and the *W1* locus, several genes and causal polymorphisms underlie the association signals. Notably, three of the markers stem from the region between 605 and 610 Mbp, two from ~ 621.5 Mbp and one from ~ 625 Mbp, with non-associated markers interspersed, which may support the latter explanation (Fig. S10). Again, future work is required to further characterize this major glaucousness locus and clone the underlying gene(s), for which this study has laid the foundation.

### Epistatic interactions contribute to the genetic control of glaucousness

**Fig. 4:**
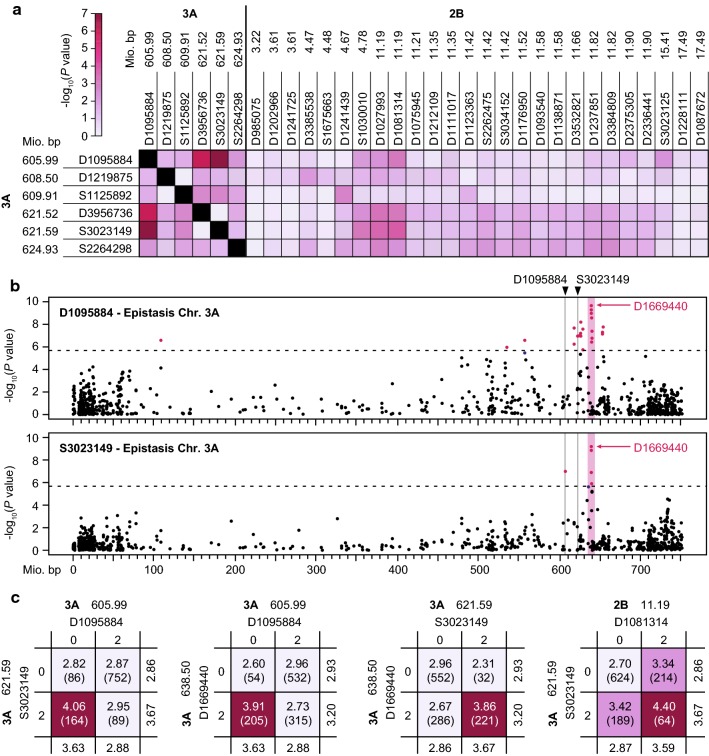
Epistatic interactions in flag leaf glaucousness. **a** Epistasis among the significantly associated markers for the two major QTL on chromosomes 3A and 2B. **b** Results from the genome-wide scan for interactions of markers D1095884 or S3023149, shown for chromosome 3A. The common region with marker D1669440 is highlighted. **c** Four-way tables showing epistatic interactions among loci. The numbers in brackets indicate the number of individuals in each genotypic class

The interactions known for the *W* and *Iw* loci prompted us to investigate epistasis among the significantly associated markers. This revealed that marker D1095884 at ~ 606 Mbp on chromosome 3A shows an epistatic interaction with the two closely linked markers at ~ 621.5 Mbp on the same chromosome (Figs. 4a, S11). Regarding interactions among markers from the 3A and 2B QTL, the strongest interaction was observed between marker S3023149 at ~ 621.5 Mbp on 3A and D1081314 at ~ 11.2 Mbp on 2B. We next focused on these markers that potentially are part of epistatic QTL and assessed them for interactions with all genome-wide markers. For both D1095884 and S3023149, this revealed an interacting locus on the same chromosome, 3A, at approximately 638.5 Mbp, represented by the most strongly associated marker D1669440 (Tables S6, S7, Figs. 4b, S12). This is reminiscent of the *W1* and *W2* loci with their *Iw1* and *Iw2* loci located close by on the same chromosomes.

Analyzing these epistatic interactions in 4-way tables revealed that the interaction between D1095884 and S3023149, as well as those between these two markers and D1669440, has three genotypic classes with comparable, low glaucousness and one genotypic class with a much higher glaucousness (Fig. 4c). Thus, within the 3A QTL, a combination of certain alleles is required for a high glaucousness, which might resemble a certain haplotype if several genes are involved. The interaction with D1669440 shows that the 3A QTL glaucousness allele will only lead to a high glaucousness in the presence of one of the alleles of this epistatic locus, whereas the other allele results in a glaucousness as low as that of the non-glaucous allele at the 3A QTL. By contrast, the nonsignificant interaction between the 3A and 2B QTL resembles an additive interaction, with the effects of the two QTL being additive and thus the lowest glaucousness for the genotypic class with the non-glaucous allele at both loci, the highest glaucousness if both glaucous alleles are present in a genotype, and an intermediate glaucousness for those carrying only one of the glaucous QTL alleles. Taken together, just as for the *W1* and *W2* loci, the 3A QTL appears to depend on epistatic interactions, illustrating the importance of epistasis in the expression of glaucousness.

## Discussion

The consequences of climate change, already witnessed now, will not only increase average temperatures, but also the occurrence of extreme weather conditions. Confronting the latter with cultivars capable of tolerating such events is a major challenge for plant breeding. Therefore, understanding the genetic basis of traits potentially related to improved stress tolerance is an important step.

Glaucousness has been implicated in conferring tolerance against abiotic stresses in small grain cereals. It is conceivable that the epicuticular wax layer can affect the plants’ physiological response to environmental stimuli, in particular to heat and drought stress. Our study revealed a large phenotypic variation for flag leaf glaucousness in a panel of wheat cultivars of worldwide origin. Surprisingly, we did not observe the expected pattern of a higher glaucousness in cultivars from the more heat and drought-prone countries of origin. It must be noted that the results reported for the effect of glaucousness on drought tolerance are ambiguous (e.g., Johnson et al. 1983; Merah et al. 2000; Simmonds et al. 2008). Johnson et al. (1983), for example, investigated near-isogenic wheat lines (NILs) that differed in the presence or absence of glaucousness. They showed that the glaucous lines yielded significantly more grain and dry matter than their non-glaucous counterparts in the higher yielding environments, but not in the dry environment. Moreover, no consistent differences were observed for leaf water potential or gas exchange of flag leaves. Thus, while the variation observed in our study among, as well as within, countries of origin might simply reflect certain preferences of the breeders, it could also indicate selection on glaucousness enforced by traits other than heat or drought stress. This observation does not mean, however, that glaucousness is not involved in conferring heat and drought tolerance. Both are complex traits, and at most, glaucousness is only one of the components. Johnson et al. (1983) also reported a higher surface reflectance of the abaxial side of glaucous flag leaves, which is the leaf side often displayed to the sun, particularly when the leaves roll in response to drought. Interestingly, the glaucous NILs did not necessarily have greater amounts of epicuticular wax, indicating that it may be variations in the strength and the composition of the wax layer that are important for stress tolerance and this may often be confounded with glaucousness or may in part even be genetically linked to it. Thus, additional work is required to disentangle the role of glaucousness and the different wax layer characteristics in conferring heat and drought tolerance in wheat.

The genetics underlying glaucousness have so far been studied in biparental mapping populations. Here, genome-wide association mapping identified two major peaks in the Manhattan plot, on chromosomes 3A and 2B. Further characterization revealed the 2B QTL to contain the *W1* locus (Hen-Avivi et al. 2016), but also to be more complex, likely comprising additional loci. Zhang et al. (2015) have recently described the *W3* locus as being also involved in β-diketone biosynthesis and linked to *W1*. Thus, one of the additional peaks identified in the 2B QTL region might be *W3*. The other two peaks could be a consequence of the LD structure and, despite being physically separated, might be markers associated with *W1* or *W3*. Alternatively, these peaks might be separate QTL, which would mean that besides *W1* and *W3*, other genes or gene clusters in that chromosomal region also contribute to glaucousness. Validation of these gene(s) and whether they belong to the same metabolic cluster or act independently requires further research. In addition to the major QTL on chromosome 2B, we identified two putative QTL on chromosomes 2A and 2D. The QTL on 2D likely corresponds to *W2*, while the identification of the putative QTL on chromosome 2A indicates that this locus may be present in a homoeologous series on all three genomes.

Moreover, we identified a major QTL on chromosome 3A, confirming previous findings from a biparental mapping population of two Australian wheat lines (Bennett et al. 2012). Thus, besides the B and D genomes, also the A genome of wheat harbors major glaucousness loci. As observed for *W1*, the locus may be genetically more complex and in addition might show an epistatic interaction with another locus located a few centiMorgan distal to it. While this requires validation, it is consistent with the picture emerging for the genetic control underlying glaucousness, with wax synthesis loci and other, often dominant-acting loci that inhibit them. Thus, the unexplained proportion of genotypic variance may in part be attributable to epistatic interactions, but as revealed by the genome-wide prediction, also to additional small- or even medium-effect QTL. Examples for the latter may be the putative QTL identified on chromosomes 6A and 7B.

Taken together, we identified several promising targets for a further characterization and cloning of the underlying genes, which can certainly be assisted by utilization of the wheat tilling population and thus by a combination of forward and reverse genetics (Krasileva et al. 2017). Collectively, this will further improve our understanding of the genetic architecture of this trait in small grain cereals, toward a targeted design of the epicuticular wax layer in plant breeding.

## Electronic supplementary material

Below is the link to the electronic supplementary material.

Supplementary material 1 (DOCX 1228 kb)

Supplementary material 2 (XLSX 3071 kb)

Supplementary material 3 (XLSX 581 kb)
