# Supplementary material for: Refining the genetic architecture of flag leaf glaucousness in wheat
Source: Theor Appl Genet. 2020 Jan 17;133(3):981–91. doi: 10.1007/s00122-019-03522-x (PMC7021748; doi:10.1007/s00122-019-03522-x)
Supplement: Supplementary file 1 — Supplementary material 1 (DOCX 1228 kb) [file 122_2019_3522_MOESM1_ESM.docx]

**Table S1.** Summary statistics for flag leaf glaucousness (FLG).

|  | FLG |
| --- | --- |
| Min | 0.67 |
| Mean | 3.05 |
| Max | 6.40 |
| $\sigma_{G}^{2}$ | 1.30** |
| $\sigma_{G\times L}^{2}$ | 0.13** |
| $\sigma_{e}^{2}$ | 0.39 |
| *h^2^* | 0.87 |

Genotypic variance ($\sigma_{G}^{2}$), genotype-by-location interaction variance ($\sigma_{G\times L}^{2}$), error variance ($\sigma_{e}^{2}$), and heritability (*h^2^*). ** significant at the 0.01 probability level.

**Table S2** Results from the genome-wide association mapping. Provided as Excel table.

**Table S3** Markers of the major QTL on chromosome 3A identified as significantly associated with flag leaf glaucousness in winter wheat. Markers were either fitted singly in a linear model to estimate the proportion of explained genotypic variance (*p_G-Single_*) or jointly in the order of their *P* values (*p_G-Joint_*).

| Marker | Chr. | Pos. (cM) | Pos. (bp)^*^ | BLAST^*^ | *P* value | *p_G-Single_* | *p_G-Joint_* | Effect | *p^$^* |
| --- | --- | --- | --- | --- | --- | --- | --- | --- | --- |
| D1095884 | 3A | 109.5^&^ | 605,990,824 | 54/54, 1e-18 | 8.0e-10 | 7.73 | 0.48 | -0.38 | 0.23 |
| D1219875 | 3A | 109.5 | 608,499,059 | 55/55, 2e-20 | 2.6e-18 | 13.39 | 13.48 | 0.53 | 0.19 |
| S1125892 | 3A | 104.6 | 609,909,609 | 69/69, 5e-28 | 3.1e-7 | 0.01 | 0.12 | -0.06 | 0.89 |
| D3956736 | 3A | 106.9^&^ | 621,515,670 | 40/40, 4e-11 | 5.1e-9 | 8.43 | 0.06 | 0.40 | 0.24 |
| S3023149 | 3A | 106.9 | 621,586,759 | 37/37, 1e-10 | 4.6e-9 | 8.74 | 0.77 | 0.41 | 0.23 |
| S2264298 | 3A | 109.3 | 624,928,879 | 69/69, 5e-28 | 1.1e-7 | 10.75 | 3.19 | -0.39 | 0.39 |

^*^ physical positions of the markers based on IWGSC RefSeq v1.0

^$^ frequency of the allele increasing glaucousness

^&^ unmapped marker placed on genetic map based on LD with mapped markers

**Table S4** Markers of the major QTL on chromosome 2B identified as significantly associated with flag leaf glaucousness in winter wheat, ordered according to their physical position. Markers were either fitted singly in a linear model to estimate the proportion of explained genotypic variance (*p_G-Single_*) or jointly in the order of their *P* values (*p_G-Joint_*).

| Marker | Chr. | Pos. (cM) | Pos. (bp)^*^ | BLAST^*^ | *P* value | *p_G-Single_* | *p_G-Joint_* | Effect | *p^$^* |
| --- | --- | --- | --- | --- | --- | --- | --- | --- | --- |
| D985075 | 2B | 9.8 | 3,224,294 | 69/69, 5e-28 | 1.4e-9 | 5.56 | 0.05 | -0.27 | 0.44 |
| D1202966 | 2B | 3.9 | 3,606,549 | 69/69, 5e-28 | 2.0e-9 | 5.51 | 0.52 | -0.27 | 0.48 |
| D1241725 | 2B | 3.9 | 3,606,552 | 56/56, 5e-21 | 5.3e-9 | 5.23 | 0.40 | -0.27 | 0.48 |
| D3385538 | 2B | 7.4^&^ | 4,472,312 | 48/48, 2e-15 | 4.7e-13 | 6.32 | 0.73 | 0.29 | 0.58 |
| S1675663 | 2B | 7.4 | 4,482,655 | 54/54, 7e-20 | 8.9e-16 | 5.89 | 5.98 | -0.28 | 0.51 |
| D1241439 | 2B | 19.1 | 4,669,667 | 69/69, 5e-28 | 8.8e-9 | 3.49 | 0.19 | -0.22 | 0.63 |
| S1030010 | 2B | 8.6^&^ | 4,780,170 | 69/69, 8e-27 | 1.1e-8 | 7.89 | 1.75 | 0.38 | 0.25 |
| D1027993 | 2B | 22.4^&^ | 11,187,595 | 67/67, 1e-25 | 2.2e-7 | 6.59 | 0.29 | -0.34 | 0.25 |
| D1081314 | 2B | 22.4^&^ | 11,188,559 | 67/69, 4e-24 | 2.1e-8 | 7.23 | 0.00 | 0.36 | 0.25 |
| D1075945 | 2B | 20.2 | 11,207,595 | 68/69, 2e-26 | 1.7e-11 | 3.70 | 0.00 | 0.23 | 0.62 |
| D1212109 | 2B | 22.4 | 11,345,143 | 57/57, 2e-21 | 4.5e-10 | 3.80 | 0.00 | 0.23 | 0.63 |
| D1111017 | 2B | 24.5 | 11,345,146 | 51/51, 3e-18 | 7.8e-10 | 3.78 | 0.03 | 0.23 | 0.63 |
| D1123363 | 2B | 39.0 | 11,416,903 | 67/67, 6e-27 | 1.9e-7 | 5.32 | 0.48 | 0.27 | 0.58 |
| S2262475 | 2B | 13.7 | 11,419,493 | 69/69, 5e-28 | 1.4e-12 | 5.09 | 0.01 | 0.27 | 0.63 |
| S3034152 | 2B | 22.4 | 11,419,490 | 37/38, 1e-9 | 5.8e-12 | 5.07 | 0.00 | -0.27 | 0.63 |
| D1176950 | 2B | 22.4 | 11,519,372 | 69/69, 5e-28 | 9.5e-12 | 4.33 | 0.00 | -0.25 | 0.65 |
| D1093540 | 2B | 22.4 | 11,575,443 | 50/50, 1e-17 | 9.9e-12 | 4.66 | 0.24 | -0.26 | 0.63 |
| D1138871 | 2B | 22.4 | 11,577,302 | 55/55, 2e-20 | 3.7e-10 | 4.76 | 0.07 | -0.26 | 0.63 |
| D3532821 | 2B | 22.4^&^ | 11,660,011 | 56/64, 1e-11 | 4.1e-13 | 4.87 | 0.01 | -0.26 | 0.63 |
| D1237851 | 2B | 37.1 | 11,818,102 | 69/69, 5e-28 | 7.1e-11 | 4.55 | 0.01 | -0.26 | 0.63 |
| D3384809 | 2B | 22.4^&^ | 11,820,675 | 44/44, 3e-13 | 3.3e-13 | 4.88 | 0.04 | -0.26 | 0.63 |
| D2375305 | 2B | 22.4 | 11,898,539 | 37/42, 3e-5 | 1.9e-11 | 8.29 | 3.82 | 0.34 | 0.58 |
| D2336441 | 2B | 22.4 | 11,898,548 | 50/51, 1e-16 | 2.8e-13 | 5.19 | 0.94 | 0.27 | 0.63 |
| S3023125 | 2B | 40.7 | 15,410,148 | 37/69, 1e-10 | 5.4e-8 | 2.17 | 0.17 | 0.38 | 0.29 |
| D1228111 | 2B | 24.7 | 17,493,486 | 64/69, 1e-22 | 3.8e-7 | 0.26 | 2.75 | -0.07 | 0.49 |
| D1087672 | 2B | 24.7 | 17,493,489 | 62/69, 2e-21 | 5.2e-7 | 0.24 | 0.09 | -0.07 | 0.49 |
| D1116323 | 2B | 7.3 | - | - | 1.2e-9 | 7.54 | 1.61 | 0.32 | 0.43 |
| D3948843 | 2B | 7.4^&^ | - | - | 1.4e-10 | 4.78 | 0.01 | -0.25 | 0.55 |
| D1212583 | 2B | 8.6 | - | - | 2.8e-9 | 2.86 | 0.01 | -0.21 | 0.67 |
| D3935718 | 2B | 8.6^&^ | - | - | 4.3e-9 | 2.76 | 0.08 | -0.21 | 0.67 |
| D3025900 | 2B | 10.6 | - | - | 1.2e-12 | 5.56 | 0.69 | 0.28 | 0.63 |
| D3944736 | 2B | 22.4^&^ | - | - | 2.3e-10 | 5.16 | 0.14 | 0.27 | 0.63 |
| D3956988 | 2B | 22.4^&^ | - | - | 4.0e-9 | 2.74 | 0.08 | -0.21 | 0.67 |
| D3959401 | 2B | 22.4^&^ | - | - | 6.4e-7 | 2.31 | 0.19 | 0.18 | 0.39 |

^*^ physical positions of the markers based on IWGSC RefSeq v1.0

^$^ frequency of the allele increasing glaucousness

^&^ unmapped marker placed on genetic map based on LD with mapped markers

**Table S5** Proportion of explained genotypic variance of the developed KASP markers for the polymorphisms in the three HYD genes, copy number variation (CNV) of TraesCS2B01G006100 and TraesCS2B01G006500, and the significantly associated markers from the *W1* region, assessed in a subset of 185 lines.

| Marker | Gene / Polymorphism | *p_G-Single_* |
| --- | --- | --- |
| D985075 |  | 4.27 |
| D1202966 |  | 5.32 |
| D1241725 |  | 4.61 |
| Ta_4131632 | TraesCS2B01G006100 and TraesCS2B01G006500: A43I | 6.95 |
| TaqMan® CNV | TraesCS2B01G006100 and TraesCS2B01G006500 CNV | 5.85 |
| D3385538 |  | 5.00 |
| S1675663 |  | 5.61 |
| Ta_4493853 | TraesCS2B01G007100: V182I | 6.27 |
| Ta_4494214 | TraesCS2B01G007100: InDel [G/-] | 6.41 |
| D1241439 |  | 3.05 |

**Table S6** Epistatic interactions of marker D1095884 with other markers identified as significantly associated with flag leaf glaucousness in winter wheat.

| Marker | Chr. | Pos. (cM) | Chr. (phys.) | Pos. (bp) | *P* value |
| --- | --- | --- | --- | --- | --- |
| D1669440 | 3A | 113.4 | 3A | 638,497,563 | 2.1e-10 |
| S1142583 | 3A | 113.2 | 3A | 638,497,566 | 5.1e-10 |
| D1058986 | 3A | 113.4 | 3A | 638,497,563 | 1.1e-09 |
| D3029153 | 3A | 113.2 | 3A | 638,715,643 | 2.8e-09 |
| D3064459 | 3A | 109.4 | 3A | 625,191,429 | 6.6e-09 |
| S1002763 | 3A | 123.4 | 3A | 652,910,203 | 1.8e-08 |
| S990692 | 3A | 106.6 | 3A | 617,340,558 | 2.1e-08 |
| D1082841 | 3A | 109.9 | 3A | 627,917,234 | 2.6e-08 |
| D1101228 |  |  |  |  | 2.8e-08 |
| D3937344 |  |  | 3A | 626,098,131 | 3.8e-08 |
| D1113723 | 3A | 113.4 | 3A | 638,995,196 | 4.1e-08 |
| D1722244 | 3A | 122.2 | 3A | 652,003,873 | 4.9e-08 |
| D3064608 | 3A | 109.4 | 3A | 625,183,360 | 6.3e-08 |
| S997396 | 3A | 123.4 | 3A | 651,997,816 | 7.3e-08 |
| D1771416 | 3A | 109.3 | 3A | 623,819,806 | 1.0e-07 |
| D3951838 |  |  |  |  | 1.1e-07 |
| S3023149 | 3A | 106.9 | 3A | 621,586,759 | 1.11e-07 |
| S1216643 | 3A | 109.3 | 3A | 625,237,502 | 1.11e-07 |
| D994622 |  |  | 7B | 44,069,200 | 1.2e-07 |
| D980238 | 3A | 113.4 | 3A | 638,995,196 | 1.7e-07 |
| S1068273 | 3A | 96.0 | 3A | 556,260,082 | 2.6e-07 |
| D3023967 | 3A | 84.0 | 3A | 108,025,232 | 2.6e-07 |
| D1371827 | 3A | 118.5 | 3A | 638,854,596 | 3.8e-07 |
| D3940579 |  |  |  |  | 5.0e-07 |
| D1319251 | 3A | 105.9 | 3A | 617,059,856 | 5.8e-07 |
| D3951109 |  |  | 7A | 79,719,197 | 6.5e-07 |
| D3939213 |  |  | 3A | 617,060,594 | 6.9e-07 |
| D1256620 |  |  | 7B | 120,867,555 | 9.6e-07 |
| S1091514 | 3A | 90.3 | 3A | 534,469,384 | 1.1e-06 |
| D3956736 |  |  | 3A | 621,515,670 | 1.3e-06 |
| S1127995 | 5B | 209.8 |  |  | 1.5e-06 |
| D1123781 | 5B | 184.0 |  |  | 1.6e-06 |
| S1250769 | 3A | 113.9 | 3A | 627,829,066 | 1.8e-06 |
| D1691739 |  |  |  |  | 2.1e-06 |

**Table S7** Epistatic interactions of marker S3023149 with other markers identified as significantly associated with flag leaf glaucousness in winter wheat.

| Marker | Chr. | Pos. (cM) | Chr. (phys.) | Pos. (bp) | *P* value |
| --- | --- | --- | --- | --- | --- |
| D1669440 | 3A | 113.4 | 3A | 638,497,563 | 6.3e-10 |
| D1058986 | 3A | 113.4 | 3A | 638,497,563 | 1.4e-09 |
| D1095884 |  |  | 3A | 605,990,824 | 1.1e-07 |
| S1142583 | 3A | 113.2 | 3A | 638,497,566 | 1.3e-07 |
| D2291317 |  |  | 5B | 680,096,337 | 2.4e-07 |
| D1106113 | 2A | 8.5 | 2A | 3,779,241 | 3.7e-07 |
| S1667354 |  |  |  |  | 4.2e-07 |
| D1287609 | 2B | 0.0 | 2B | 527,482 | 1.1e-06 |
| D3029153 | 3A | 113.2 | 3A | 638,715,643 | 1.2e-06 |

**Table S8** Summary of primers and used for sequencing the three HYD genes and genotyping with the established KASP markers. Provided as Excel file.


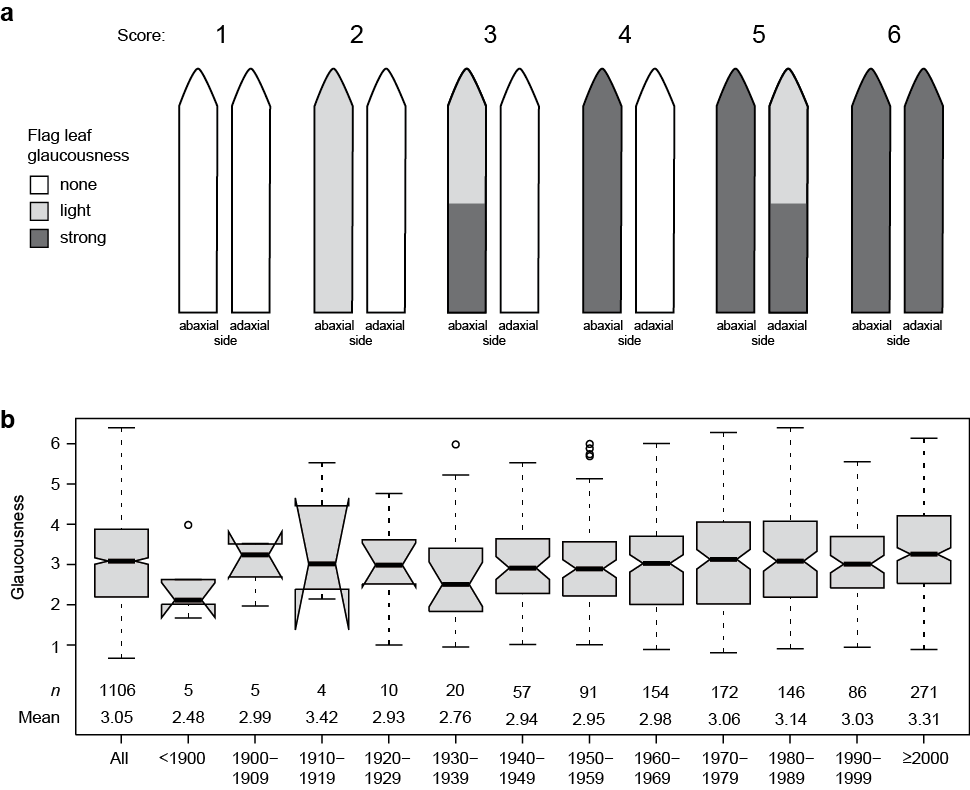


**Fig. S1** **a** Score of flag leaf glaucousness on a 1 - 6 scale. **b** Boxplots showing glaucousness dependent on the cultivars’ year of registration.


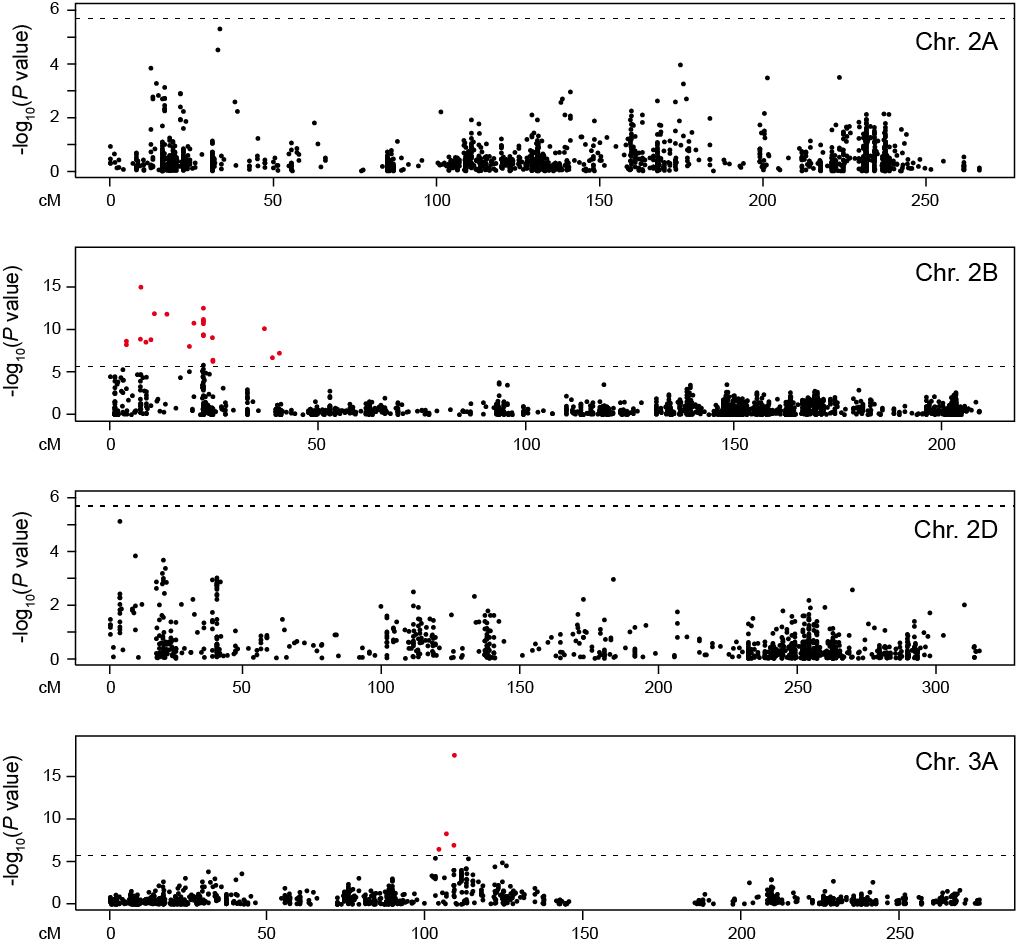


**Fig. S2** Manhattan plots of selected chromosomes based on the genetic map positions of the markers. The dashed horizontal line indicates the significance threshold (Bonferroni-corrected *P* < 0.05).


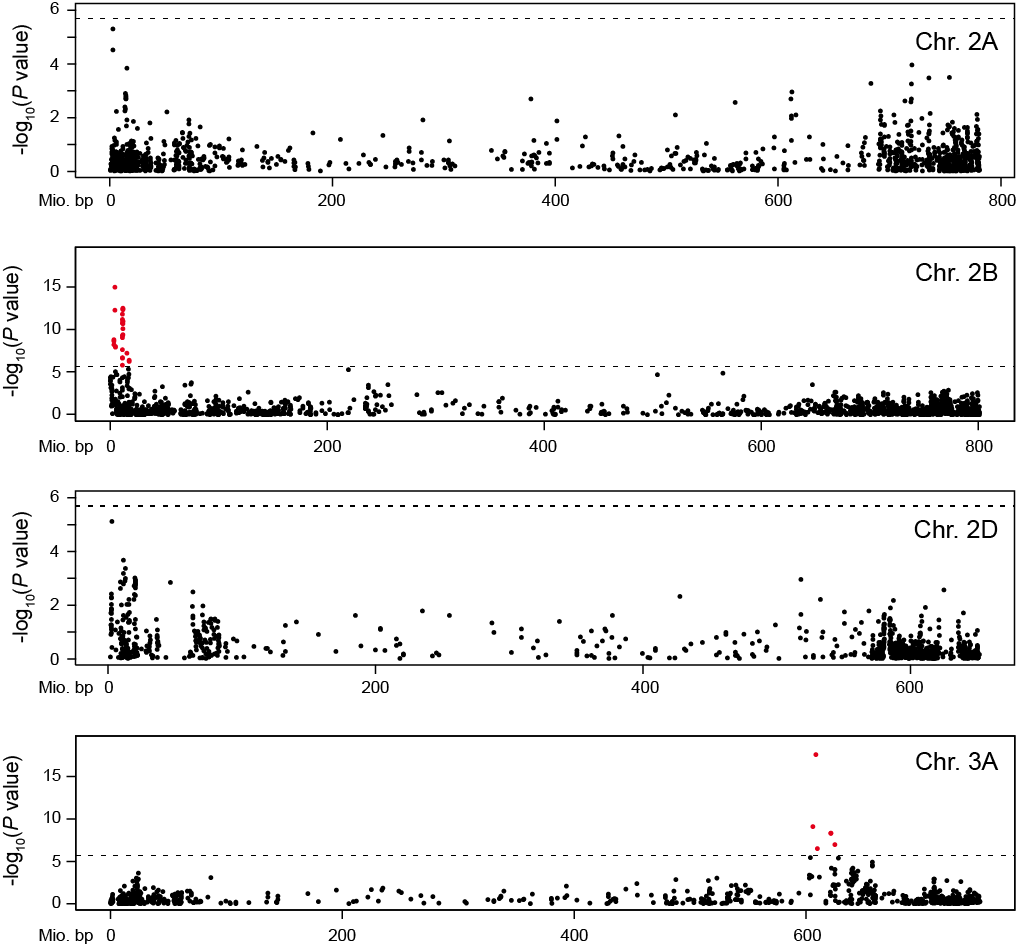


**Fig. S3** Manhattan plots of selected chromosomes based on the physical positions of the markers (based on IWGSC RefSeq v1.0). The dashed horizontal line indicates the significance threshold (Bonferroni-corrected *P* < 0.05).


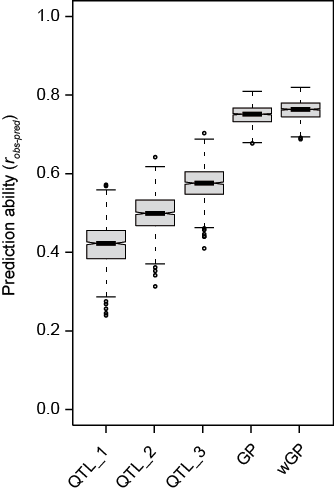


**Fig. S4** Prediction of flag leaf glaucousness. Boxplots showing the prediction ability based on the two identified QTL on chromosomes 3A and 2B (QTL_1; D1219875 and S1675663), based on these two QTL plus the putative QTL on chromosomes 2A and 2D (QTL_2; D1219875, S1675663, D2258266, and D1113129), or based on the two QTL plus the four additional putative QTL (see Table 1) (QTL_3), compared to genomic prediction (GP), or a weighted genomic prediction (wGP) that incorporates the two QTL markers as fixed effects.


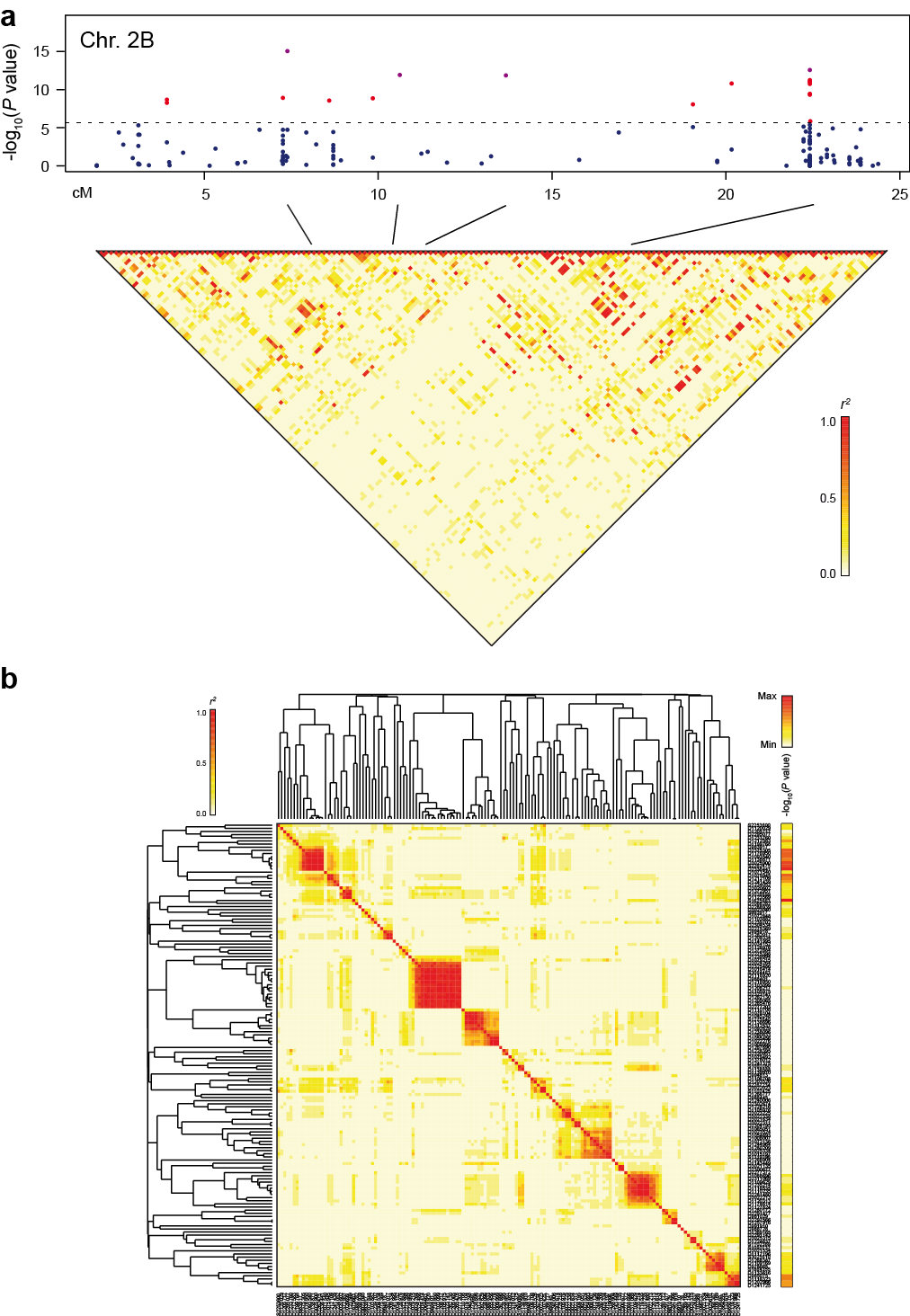


**Fig. S5** Flag leaf glaucousness QTL on chromosome 2B. **a** Manhattan plot and linkage disequilibrium (*r^2^*) pattern in the QTL region. The position in the LD plot is indicated for four significant markers colored purple. **b** Clustering of the same markers based on their linkage disequilibrium.


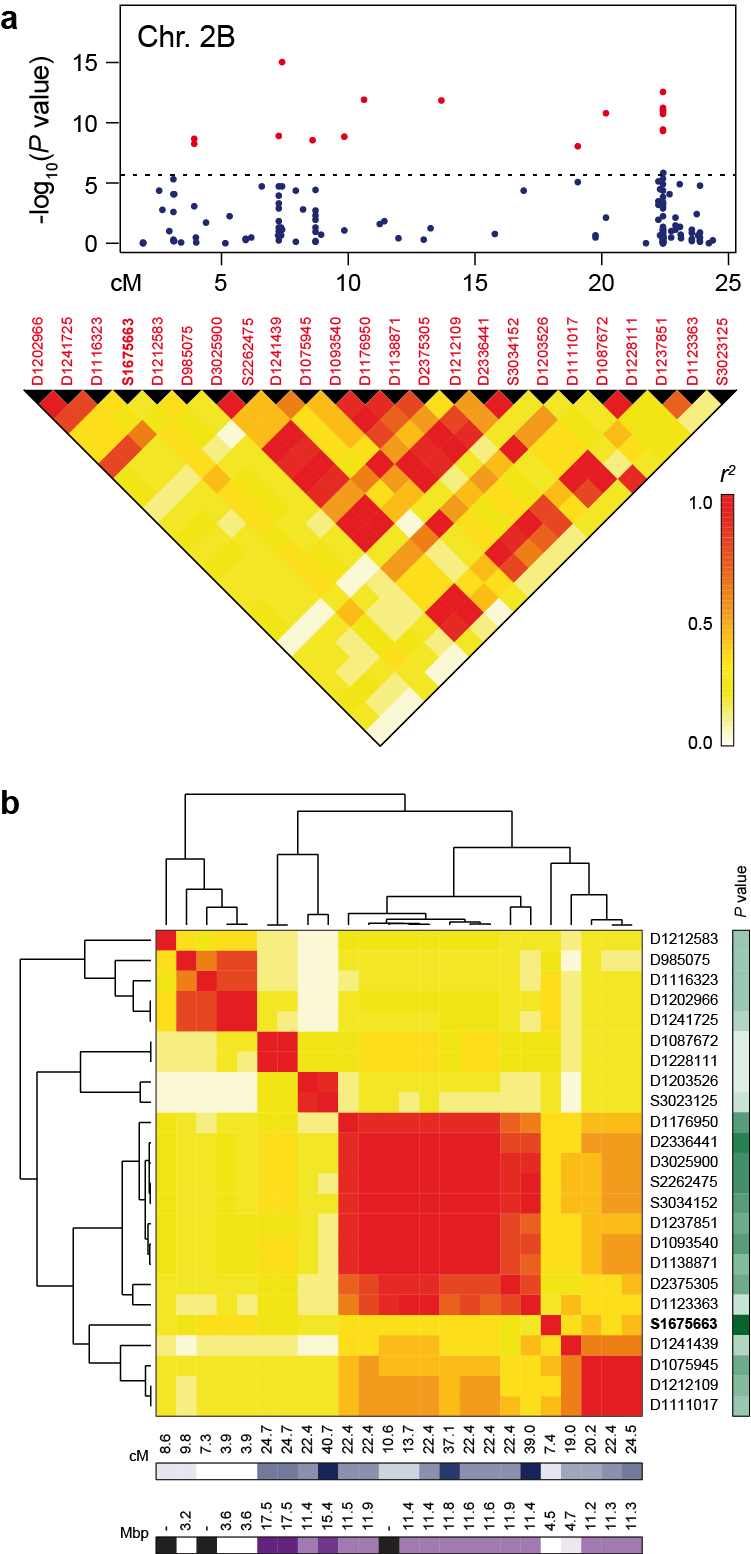


**Fig. S6** Flag leaf glaucousness QTL on chromosome 2B. **a** Manhattan plot and linkage disequilibrium (*r^2^*) pattern of the significantly associated markers. **b** Clustering of the same markers based on their linkage disequilibrium.


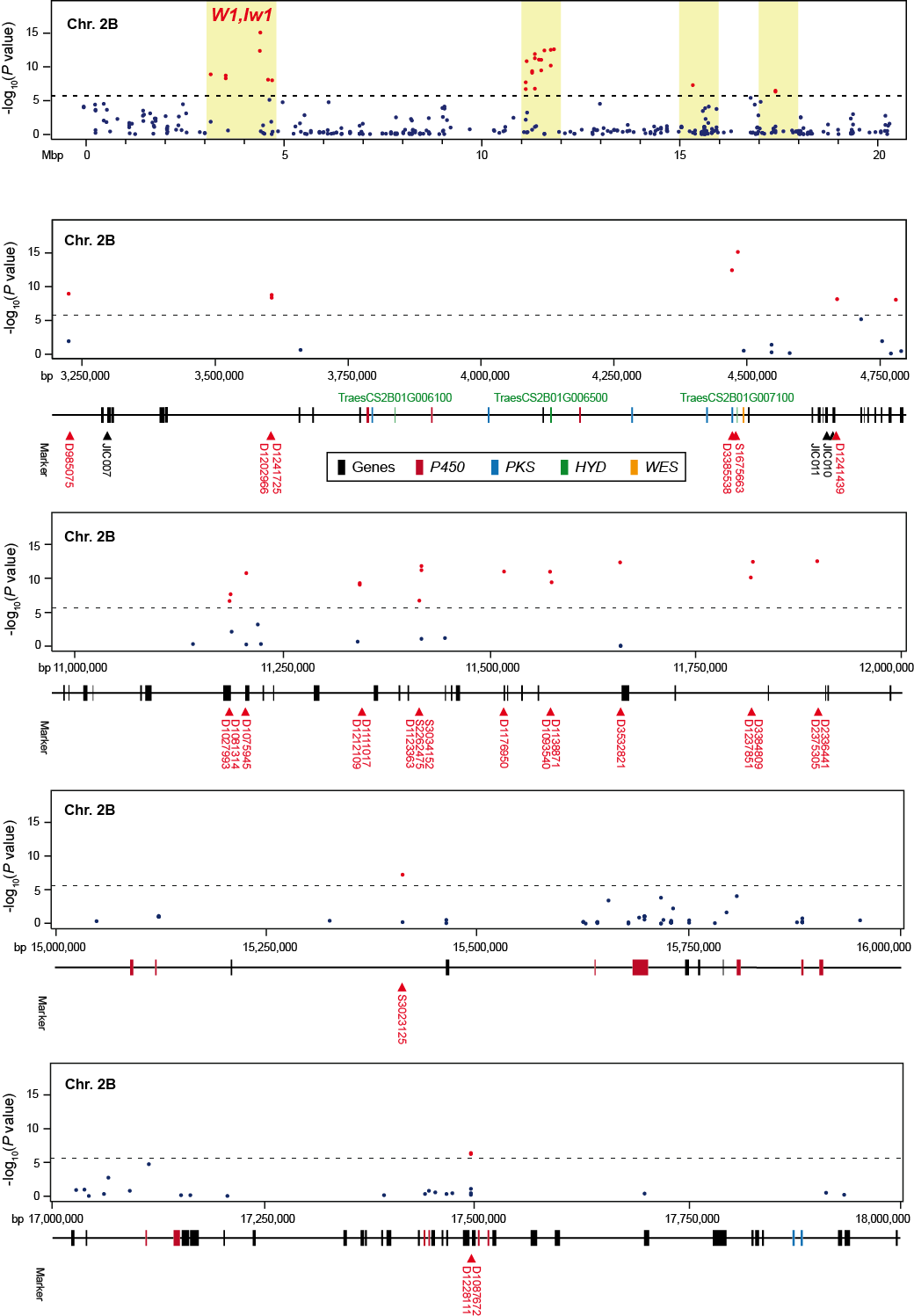


**Fig. S7** Fine-mapping of the major QTL on chromosome 2B.


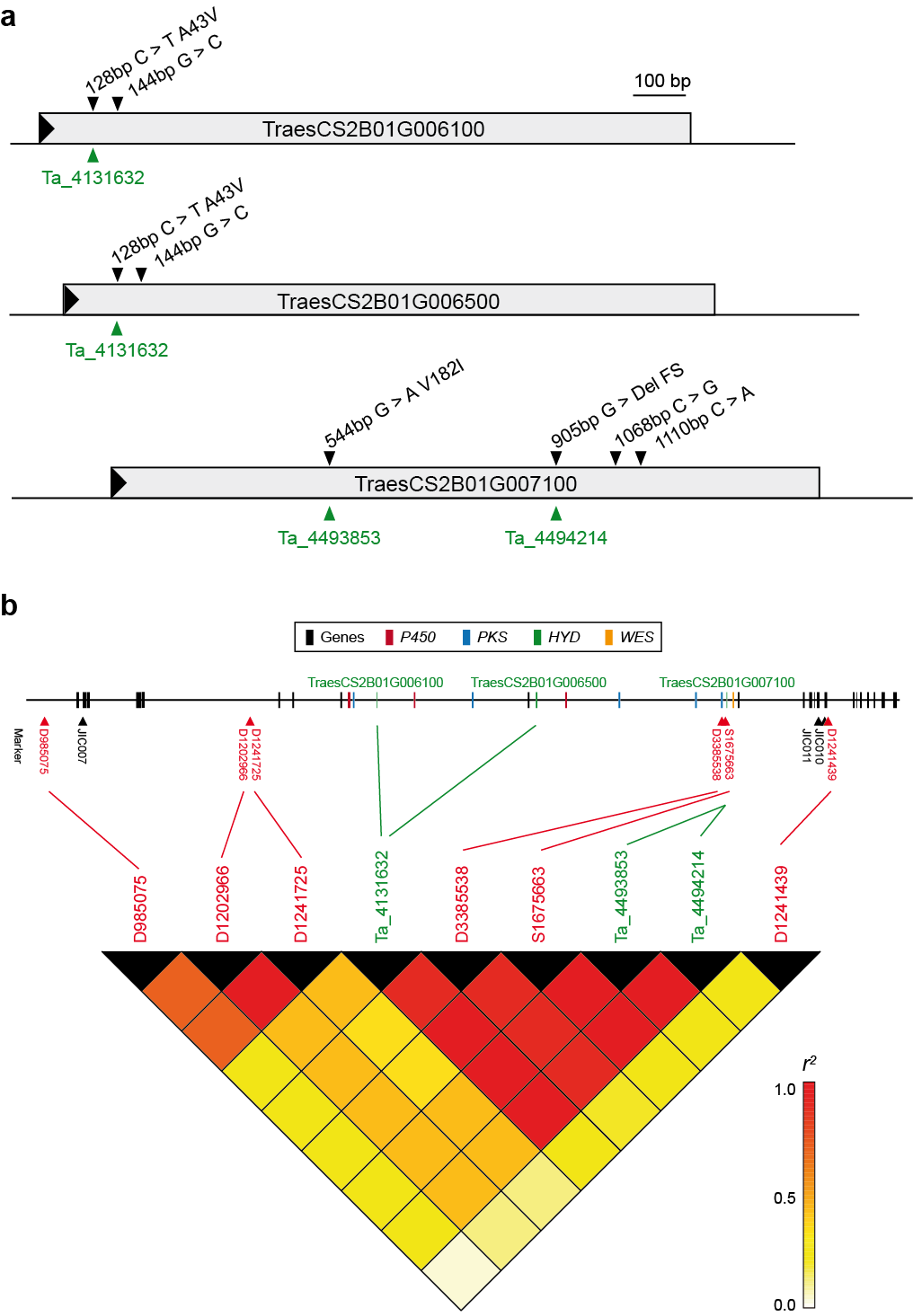


**Fig. S8** Analysis of the HYD genes. **a** Polymorphisms in the three HYD genes and the KASP markers developed for the four non-synonymous polymorphisms. **b** Linkage disequilibrium between the four KASP markers and the significantly associated markers from the *W1* region, based on genotyping a subset of 185 lines.


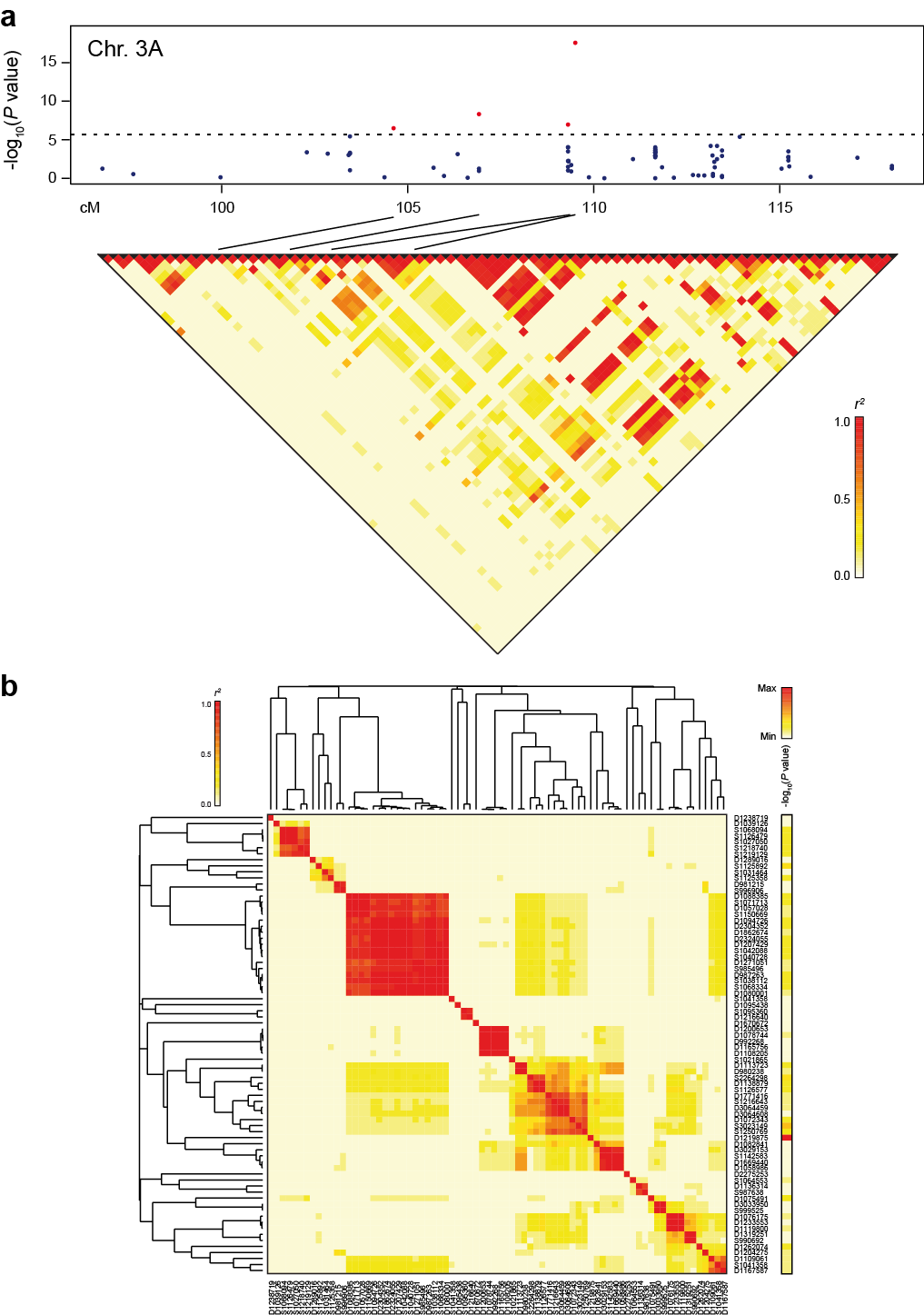


**Fig. S9** Flag leaf glaucousness QTL on chromosome 3A. **a** Manhattan plot and linkage disequilibrium (*r^2^*) pattern in the QTL region. **b** Clustering of the same markers based on their linkage disequilibrium.


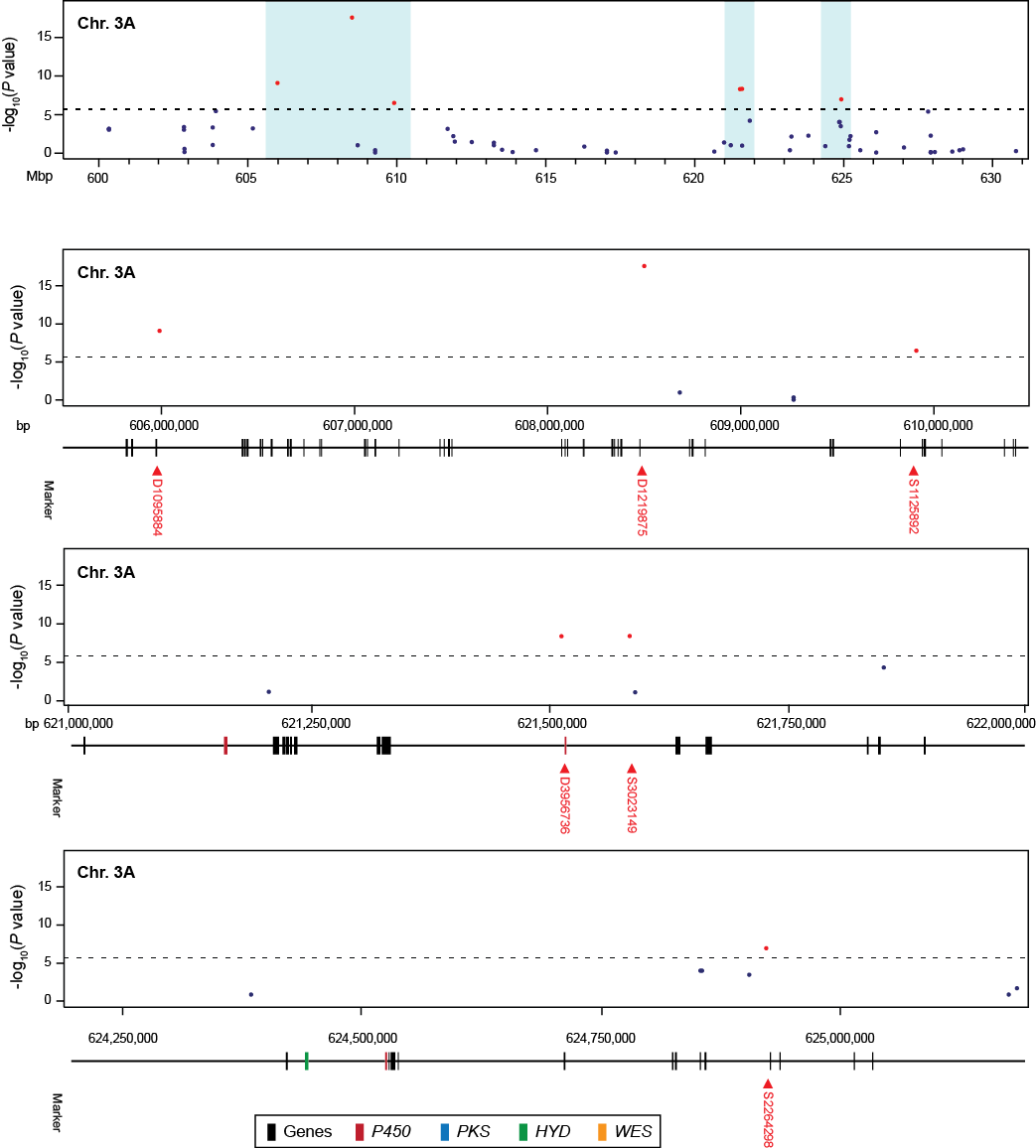


**Fig. S10** Fine-mapping of the major QTL on chromosome 3A.


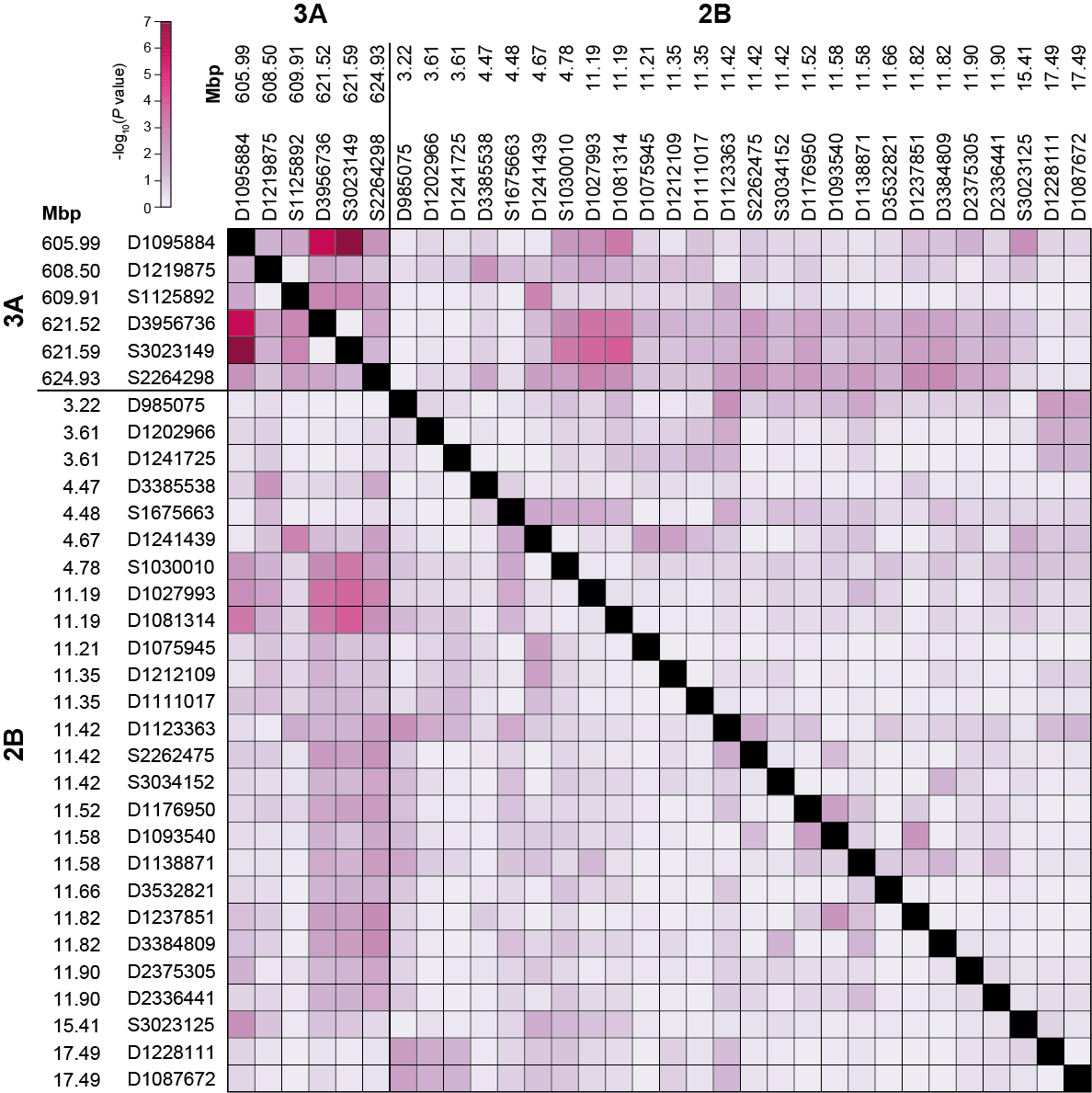


**Fig. S11** Epistatic interactions among the significantly associated markers on chromosomes 3A and 2B.


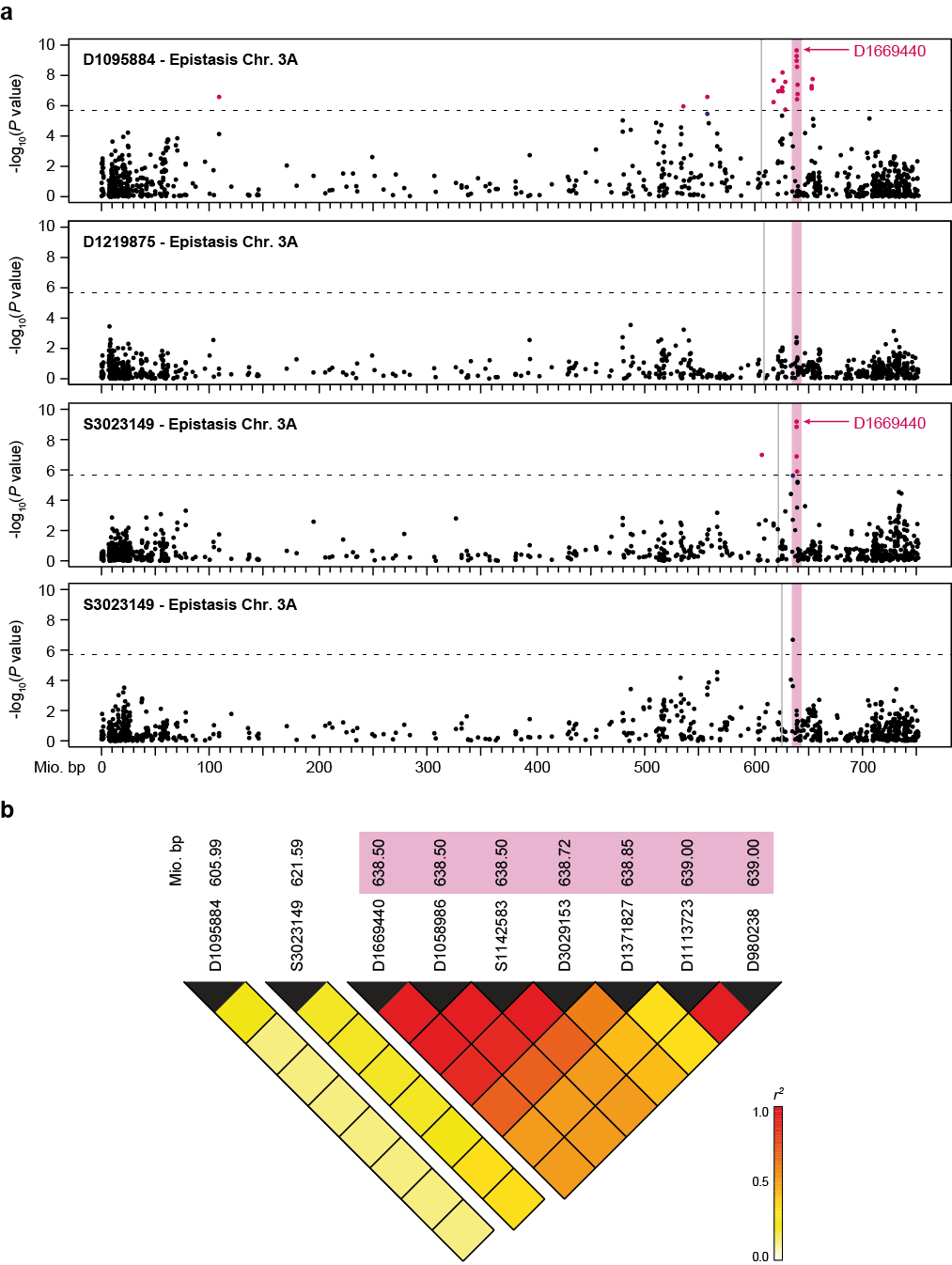


**Fig. S12** **a** Epistatic interactions among significantly associated markers on chromosomes 3A and all other markers on 3A. **b** Linkage disequilibrium among these markers.


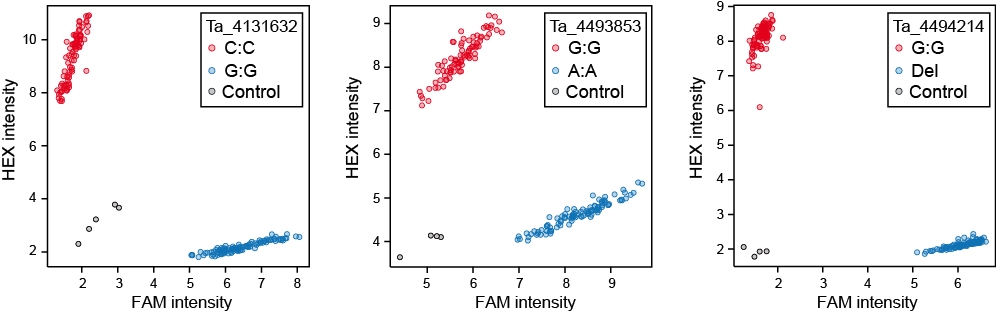


**Fig. S13** Development of KASP markers for the identified polymorphisms in the three HYD genes.
